# Resilience and Quality of Life in Breast Cancer Patients

**DOI:** 10.3889/oamjms.2015.128

**Published:** 2015-12-08

**Authors:** Gordana Ristevska-Dimitrovska, Izabela Filov, Domnika Rajchanovska, Petar Stefanovski, Beti Dejanova

**Affiliations:** 1 *University St. Kliment Ohridski Bitola, Higher Medical School Bitola, Bitola, Republic of Macedonia*; 2 *Clinical Hospital Dr. Trifun Panovski, Department of Oncology, Bitola, Republic of Macedonia*; 3 *Department of Medical and Experimental Physiology with Anthropology, Medical Faculty, Ss Cyril and Methodius University of Skopje, Skopje, Republic of Macedonia*

**Keywords:** resilience, quality of life, breast cancer, Connor Davidson Resilience Scale, EORTC QLQ-C30

## Abstract

**BACKGROUND:**

Many studies have shown that a relationship exists between quality of life (QoL) and resilience in breast cancer patients, but few studies present information on the nature of this relationship of resilience on QoL. Our aim was to examine the relationship between resilience and quality of life in breast cancer patients.

**METHODS:**

QoL was measured in 218 consequent breast cancer patients, with EORTC - QLQ Core 30 questionnaire, and EORTC QLQ-BR23. The resilience was measured with Connor Davidson Resilience Scale.

**RESULTS:**

The global quality of life was positively correlated with the levels of resilience (R = 0.39 p < 0.001). All functional scales (physical, role, emotional, cognitive and social functioning was in a positive correlation with resilience. The symptoms severity (fatigue, nausea and vomitus, pain, dyspnea, insomnia, appetite loss, constipation, diarrhea, financial difficulties) was in negative correlation with resilience. Less resilient breast cancer patients reported worse body image and future perspective and suffered from more severe adverse effects of systemic therapy, and arm/breast symptoms.

**CONCLUSION:**

Our findings show that psychological resilience affects different aspects of health-related quality of life. More resilient patients have significantly better quality of life in almost all aspects of QoL.

## Introduction

### Resilience

Psychological resilience represents an ability of a person to protect his/her mental health when faced with adversity such is the cancer diagnosis. Adversity, through resilience mechanisms, can be reframed and become a possibility that one can learn and even benefit from. Psychiatry, as well as psychology, have repeatedly addressed the negative outcomes derived from cancer experience, such as depression, post-traumatic stress symptoms as well as anxiety [1]. But, very little is known about, what drives people to fight, survive and grow, when faced with cancer. When describing the cancer experience, one can approach resilience from several aspects [2]. We can approach to resilience as a baseline characteristic, a personality trait that enables individual to maintain mental health when exposed to stressful situation [3, 4]. People who are full of optimism, hope, are motivated and have solid social support system prior to the adversity, may be considered as more resilient [5, 6]. Second way of understanding resilience is to view it as a mechanism, which a person can use to adapt and protect good mental health [7]. Another way of understanding resilience is if we measure it as an outcome, as a consequence of adversity [8]. When resilience represents consequence, there is no way for an individual to know if he/she is resilient until faced with adversity.

Cancer survivors, who are more optimistic and hopeful for the future, cope better with cancer and even experience personal growth [9-13]. We can expect that breast cancer survivors, who are married [14, 15], have bigger and better social support system [16-18], and have positive coping styles [19], to be more resilient and experience more growth as benefit from cancer diagnosis.

Resilience can change and modify over time, is affected by many different situations and adversities that a person overcomes in a lifetime. Resilience represents a newer concept, which deserves to be targeted from the beginning of life with cancer. Clinicians should spend more time and effort to create interventions that enable patients to foster their resilience [2].

### Quality of life

Cancer medicine has become increasingly effective, so the attention has shifted to issues surrounding cancer survivorship. The central challenge of cancer is still focused on being cancer free, but it is more and more important to maintain and improve the patients’ quality of life [20]. When the concept of quality of life in cancer care emerged in the 1980’s, it was more understood as an issue of functionality and ability to effectively execute regular daily activities [21]. In time, the concept became broader and more aspects became integral part of contemporary QoL measurement, such as cognitive, emotional, social and sexual functioning.

Many different factors influence quality of live in cancer patients and survivors. Individuals view the world from very different angles and they differ in their emotional and behavioural responses to adversity. Individual differences of cancer patients/survivors have important role in quality of life, in part because quality of life is shaped by some personality traits, not only by physical, socio-demographic and oncological variables [20].

Understandably, the psychological distress that follows cancer diagnosis and treatment, negatively affects psychological components of QoL, in the beginning of cancer experience. What is not sufficiently explored is how much the initial psychological distress is influencing QoL of patients who are cancer survivors. Also the long-term side-effects of breast cancer treatments on QoL are not conclusively confirmed nor dismissed. Is there a possibility that some psychological problems surrounding cancer become permanent in time? [22]. The future research should address these dilemmas and potentially highlight the individuals at risk.

Most of the QoL research in cancer patients is focused on variables that negatively affect QoL domains (psychological distress, anxiety, depression, cognitive decline, insomnia, fatigue and other physical symptoms). The management of symptoms and disorders should occupy high position in the priority list, but it is also important to shift the focus from what declines QoL to what improves QoL, and do some more research on ways to help strengthen patients’ resilience mechanisms.

The aim of this study was to explore the association and the effect of resilience on health-related quality of life in breast cancer patients.

## Methods

### Patients

Patients from the oncology department of the Clinical Hospital “Dr. Trifun Panovski” in Bitola and University Clinic for Oncology and Radiotherapy in Skopje were included in this study. Participation in the study was offered to all patients who came for follow-up in the oncology wards in Bitola and Skopje and 218 patients met the inclusion criteria. All participants are women treated for breast cancer, older than 18 years, treated with different oncological therapies and are in a different time period from the initial cancer treatment. We used the following inclusion criteria: (1) women, aged between 18 and 90 years; (2) literate and fluent in Macedonian language; (3) no history for prior mental disorder; (4) histopathologically confirmed diagnosis of early breast cancer; (5) completed the initial surgical and oncological treatment at least 1 month prior the inclusion. All participants have given written informed consent for participation in the study. The study was approved by The Ethics Committee for research on people at the Medical Faculty, University “St. Cyril and Methodius” in Skopje.

### Procedures

All patients answered a questionnaire for psychosocial and oncological data specifically designed for the study. Some oncological parameters were collected from the medical data in the oncological wards. The psychological resilience was measured with Connor-Davidson Resilience Scale 25 (CD-RISC25) [23, 24], a self-evaluation instrument with 25 items that are ranked on a Likert scale from 0 to 4 points. The full range of the scale is 0-100 points, where higher scores indicate higher resilience.

Health-related quality of life (HRQoL) was assessed with the Macedonian version of EORTC core quality of life questionnaire (EORTC QLQ-C30, version 3 together with the breast cancer module EORTC QLQ-BR23), which measures different aspects of quality of life relevant to patients with cancer. The EORTC QLQ-C30 questionnaire includes five functional scales (physical, role, cognitive, social, and emotional), nine symptom scales (nausea, pain, fatigue, dyspnoea, diarrhoea, constipation, insomnia, appetite loss, financial difficulties), and a global health scale. All scales range in score from 0 to 100. The EORTC QLQ-BR23 focuses on systemic therapy side effects, arm and breast symptoms, and includes several sexual items. For functional scales, higher scores indicate better levels of functioning and/or better quality of life, while in symptom scales higher scores indicate more severe symptoms [25]. The sexual items were excluded from this analysis, due to small amount of valid data.

## Results

In total, 218 participants, women with early breast cancer at an average age of 60.2 years (range 30-90), were included in this study. The percentage of participants with primary school, high school, and university education levels were 25.8%, 40.6%, and 33.6%, respectively. Almost half of the patients 106 (49.5%) were retired, 58 (27.1%) were employed and 50 (23.4%) were unemployed. Among the 218 participants, 52 (27.9%) had I stage breast cancer, 74 (39.8%) had second stage, and 60 (32.3%) had third stage breast cancer. Moreover, 72 (33%) patients underwent surgery combined with chemotherapy, 11 (5%) underwent surgery combined with radiotherapy, 104 (47.7%) underwent surgery combined with chemotherapy and radiotherapy, and 31 (14.2%) underwent only surgery. Most of the surgically treated patients underwent mastectomy 165 (77.8%), followed by lumpectomy 40 (18.9%) and double mastectomy 7 (3.3%). In the moment of the interview 206 patients gave valid information about the adjuvant treatment: 134 patients (65%) receive adjuvant therapy (tamoxifen 89.2% or aromatase inhibitors 10.8%). Most of the patients 86 (40.8%) were cancer free women for more that 5 years since initial diagnosis, 74 (35.1%) were diagnosed 2-5 years ago, 28 (13.2%) were diagnosed 1-2 years ago, and 23 (10.9%) were diagnosed 6 months-1 year ago.

The global quality of life was positively correlated with levels of resilience (R = 0.39, p < 0.001). All functional scales were in positive correlation with resilience: physical functioning (R = 0.3, p < 0.01), role functioning (R = 0.27, p < 0.01), emotional functioning (R = 0.49, p < 0.001), cognitive functioning (R = 0.39, p < 0.001) and social functioning (R = 0.4, p < 0.001).

**Figure 1:**
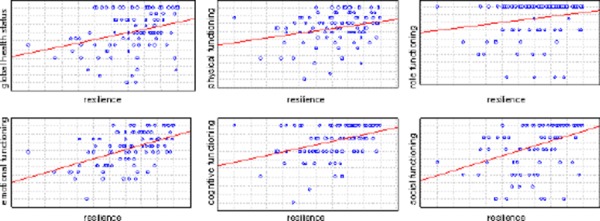
*Correlation between global health/functioning scales and resilience*.

When we analyzed the symptoms and the resilience we found that more resilient patients report less severe symptoms. The symptom severity is in negative correlation with resilience: fatigue (R = - 0.363, p < 0.001), nausea and vomitus (R = - 0.3, p = 0.000006), pain (R = - 0.3, p < 0.01), dyspnea (R = - 0.29, p < 0.01), insomnia (R = - 0.352, p < 0.01), appetite loss (R = - 0.474, p < 0.001), constipation (R = - 0.217, p < 0.01), diarrhea (R = - 0.182, p < 0.01), financial difficulties (R = - 0.176, p < 0.01).

**Figure 2:**
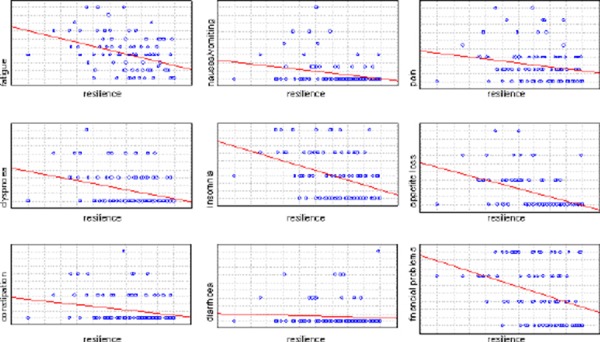
*Correlation between symptom severity and resilience*.

Less resilient breast cancer patients reported worse body image (R = 0.35, p < 0.001) and future perspective (R = 0.369, p < 0.001). Also, less resilient patients suffered from more severe adverse effects of systemic therapy (R = - 0.366, p < 0.001), symptoms from arm (R = - 0.198, p < 0.01) and breast (R = - 0.287, p < 0.01), as well as hair loss (R = - 0.3, p < 0.01).

**Figure 3:**
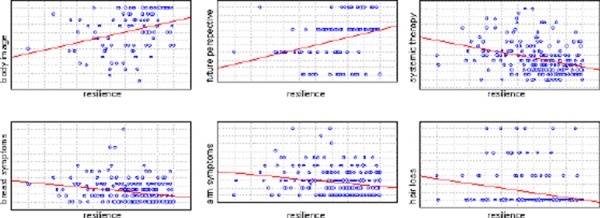
*Correlation between body image, future perspective, systemic therapy symptoms, breast/arm symptoms, hair loss and resilience*.

## Discussion

Although a lot of research has shown that a resilience affects quality of life in cancer patients, data about the direction and the degree of this influence is not conclusive. The data obtained by our observational study showed that resilience is a major influencing factor in almost all aspects of QoL.

The global quality of life was positively correlated with levels of resilience. All functional scales were in strong positive correlation with resilience. Emotional, social, cognitive and role functioning were significantly better in more resilient patients. When we analyzed the symptoms and the resilience we found that more resilient patients report less severe symptoms. The symptom severity (fatigue, nausea and vomitus, pain, dyspnea, insomnia, appetite loss, constipation, diarrhea, financial difficulties) is in strong negative correlation with resilience. Less resilient breast cancer patients, as opposed to more resilient patients, had worse body image and their outlook on life was more pessimistic. Also, in less resilient patients physical functioning was worse and they had more severe adverse effects of systemic therapy and arm/breast symptoms.

Beyond doubt, we cannot determine the direction of the influence of resilience and QoL on each other based on our data. It would be an over-simplification of the complex psychological life of the cancer patients, to assume that QoL is affected only by the patients’ psychological resilience. A lot of other psycho-social and oncological variables affect QoL. Their complex interplay should be taken into account.

Nevertheless, resilience as a stable individual resource, has its own role into QoL improvement, that should be taken into account. Through analyzing the QoL of patients with cancer, we should select the most effective strategies that can improve patients’ resilience [26]. The continued development of individual and group-based interventions that support positive outcomes and more functional coping styles [27] may be valuable and contribute to our understanding of resilience among cancer survivors. Our investigation shows that patients who are less depressed have higher levels of resilience and that psychological resilience may independently contribute to lower levels of depression among breast cancer patients. The level of psychological resilience may be a protective factor for depression and psychological distress [28]. Through endorsing patient’s strengths, we can help the increase of quality of life.

In conclusion, our findings show that psychological resilience affects different aspects of health-related quality of life. More resilient patients have significantly better quality of life in almost all aspects of QoL. These results can be used as a base for development of psychosocial interventions that will target psychological resilience in cancer patients.
